# The effect of simulation training on midwifery students’ skills, satisfaction with learning, anxiety, and self-efficacy in neonatal heel prick blood collection

**DOI:** 10.1186/s12909-025-08303-3

**Published:** 2025-12-27

**Authors:** Seval Cambaz Ulaş, Seçil Köken Durgun, Yonca Çi̇çek Okuyan

**Affiliations:** Faculty of Health Sciences, Department of Midwifery, Manisa Celal Bayar University, Yunusemre, Manisa, 45030 Türkiye

**Keywords:** Skills training, High-fidelity simulation environment, Facilator, Midwifery education

## Abstract

**Background:**

Simulation is often used in midwifery education because it enables students to practice clinical skills in a safe environment where mistakes can occur without the risk of person harm. This study was performed to measure the effect of a high-fidelity simulation environment and facilitator providing support through a panel on midwifery students’ skills, satisfaction with learning, anxiety, and self-efficacy in performing neonatal heel prick blood collection.

**Methods:**

The study is quasi-experimental desıgn. The study sample consisted of 92 students in total, with *n* = 46 in the control group and *n* = 46 in the experimental group (*N* = 92). The control group was instructed and evaluated on neonatal heel prick blood collection skills using a structured scenario in a simulation laboratory, with face to face facilitator support and without the use of a control panel. The experimental group underwent the same instructional and testing procedures using the same simulation scenario in a high-fidelity simulation environment, where facilitator support was provided remotely via a control panel.

**Results:**

The study revealed that students’ neonatal heel prick blood collection skills were similar and were not affected by the high-fidelity simulation environment. The mean scores for the State-Trait Anxiety Inventory (STAI) and the Student Satisfaction and Confidence in Learning Scale (SCLS) were significantly higher in the high-fidelity simulation environment (*p* < 0.05). No statistically significant difference was found in the Self-Efficacy-Sufficiency Scale (SESS) scores between students in the experimental and control groups (*p* > 0.05).

**Conclusion:**

This study found that students’ heel prick skills and self-efficacy were similar regardless of the fidelity level of the simulation laboratories and the role of the facilitator. In cases where the high-fidelity simulation environment and the facilitator provided support through a panel, students’ anxiety and satisfaction levels were higher.

**Supplementary Information:**

The online version contains supplementary material available at 10.1186/s12909-025-08303-3.

## Background

Simulation-based education is a robust, evidence-backed teaching strategy in midwifery education [1]. It involves targeted role-playing, enabling students to practice various skills [2]. Midwifery students generally view simulation-based education as essential and believe more exposure to it is crucial for their preparation [3]. Simulations are categorized by fidelity in three categories: low, medium, and high [4]. High-fidelity simulation (HSF), environments typically consist of a control room and a practice room designed to mimic a hospital setting, where students can simulate real patient care scenarios under instructor supervision. Practice rooms enable students to experience the feeling of practicing with a real patient in a real patient room. HSF often employed in healthcare training, has been shown to enhance student skills [5]. Recent regional studies also support the effectiveness of high-fidelity simulation in improving nursing students’ self-satisfaction, confidence, and practical skills. Toqan et al. [6] found that high-fidelity simulation significantly enhanced students’ self-satisfaction and self-confidence in nursing education. Similarly, Hodrob et al. [7] demonstrated improved performance and satisfaction following a high-fidelity airway management simulation in Palestine. Moreover, Jawabreh et al. [8] confirmed the effectiveness of HFS in mental health nursing education, showing positive effects on students’ practice, satisfaction, and confidence. These studies underscore the growing evidence that HFS contributes to experiential learning and self-assurance in clinical practice, aligning with the current study’s focus on neonatal skill acquisition in midwifery education.

Midwifery education integrates both theoretical learning and clinical practice [9]. Students often experience anxiety and stress when entering new clinical environments [1]. Such stress can hinder students’ ability to apply theoretical knowledge, leading to fear of mistakes and feelings of professional inadequacy [4].

The heel prick procedure is widely used as one of the basic newborn screening tests in North America and Europe [10]. In Turkey, neonatal heel prick blood collection is performed as part of a national screening program for inherited metabolic disorders, including phenylketonuria, hypothyroidism, biotinidase deficiency, cystic fibrosis, and spinal muscular atrophy (SMA) [11]. This procedure is conducted twice, first within 48 h of birth in a hospital setting, and again within the first five days at family health centers [12]. As part of their preventive health services, midwives are responsible for neonatal heel prick blood collection [13]. A study has indicated that although newborns experience pain during heel pricks, healthcare professionals often fail to recognize it, underscoring the importance of minimizing procedures [14]. Training midwives in simulation, before they develop full competence in neonatal heel prick blood collection, can help reduce the number of procedures and potential errors [15-17]. In midwifery, simulation-based education supports experiential learning, reducing anxiety and boosting both learning satisfaction and self-efficacy [18-20]. This study seeks to evaluate the effects of simulation training on midwifery students’ skills in neonatal heel prick blood collection, along with their learning satisfaction, anxiety, and self-efficacy.

## Methods

### Study design

The research was quasi-experimental desıgn.

### Place and duration of the research

The population of the study consisted of Department of Midwifery students at *********, Faculty of Health Sciences. Data for the experimental group were collected in a high-fidelity simulation environment with a remote/panel-based facilitator, while data for the control group were collected in a simulation laboratory with face-to-face facilitator support. We treated the simulation environment and the role of the facilitator as a simultaneous intervention. A simulation laboratory is a laboratory where the hospital environment (bed, woman model, baby bed, baby model) is simulated. The trainer can stand next to the student and monitor the student. A high-fidelity simulation environment consists of a simulated hospital room (bed, woman model, baby bed, baby model, radiant heater, midwife table, shelf, monitor, bedside oxygen systems, treatment cart) and a control room where instructors manage the scenario and observe/record the practice. Through this control room, instructors can monitor the student in the simulated hospital room.

### Setting and participants

**Fig. 1:**
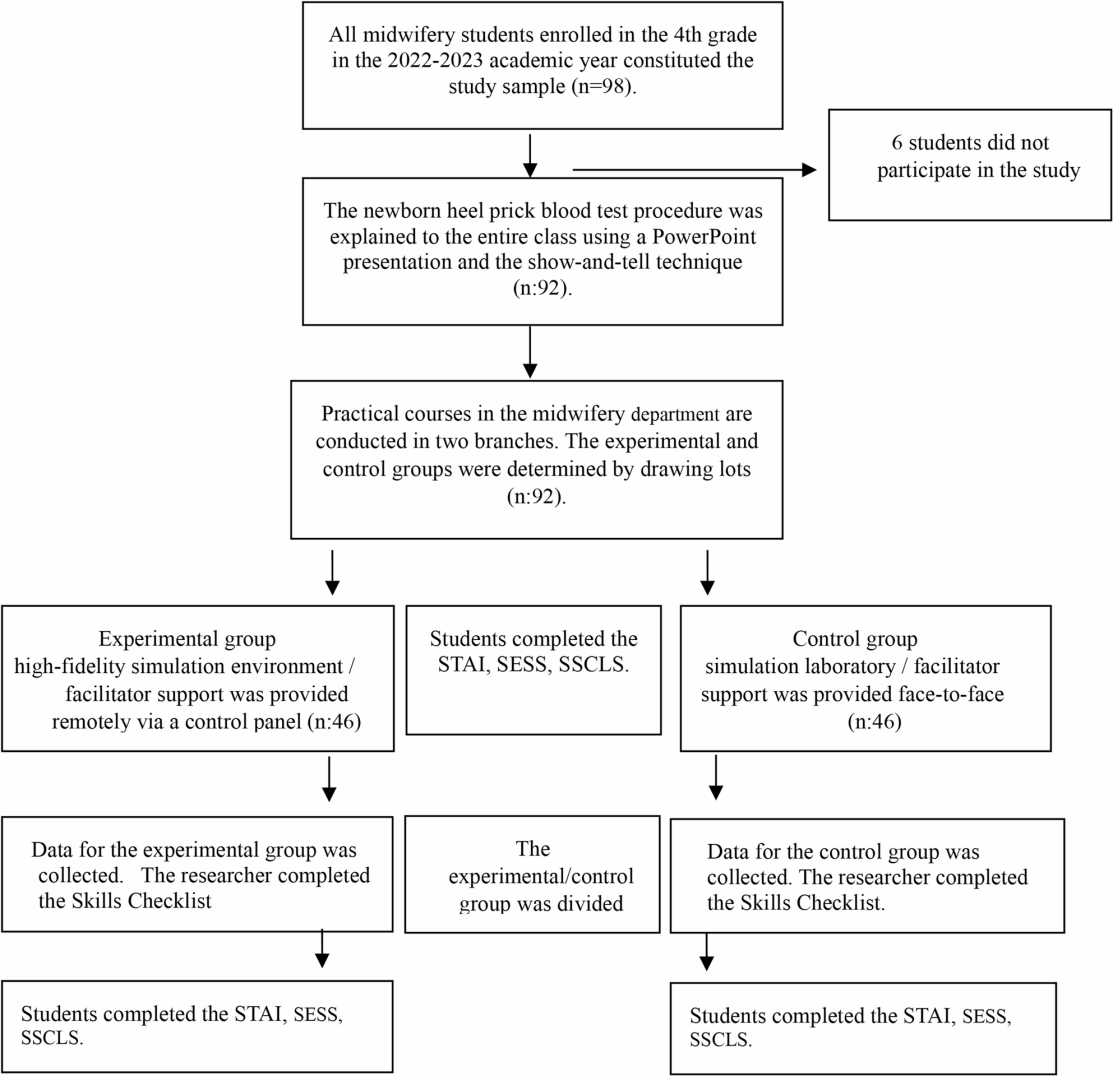
Research Flow Chart

No sample selection method was used in the study. All midwifery students enrolled in the 4th grade in the 2022–2023 academic year constituted the study sample (n: 98). Practical courses in the midwifery department are conducted in two branch. These sections are determined by the Student Affairs Office according to the last two digits of the students’ numbers (Odd numbers/Branch A, Even numbers/Branch B). In this study, the researchers determined which section would represent which simulation group by drawing lots. In this study, the researchers determined which branch would represent which simulation group by drawing lots. Accordingly, the Branch A (Experimental group, *n* = 49) was assigned to a high-fidelity simulation environment where facilitator support was provided remotely via a control panel, while the Branch B (Control group, *n* = 49) was assigned to a simulation laboratory where facilitator support was provided face-to-face. At the beginning of the study, two students who wished to withdraw, two students who suspended their enrolment, one student with poor attendance, and one student with a health issue were excluded from the research. The study was completed with a total of 92 students, comprising the high-fidelity simulation environment (*n* = 46) and the simulation laboratory (*n* = 46), (Fig. 1).

### Interventions

Stage 1: Before collecting the research data, the steps of the neonatal heel prick blood collection procedure were explained to the entire class using a PowerPoint presentation and a show-and-tell technique in the classroom. The training covered the introduction of the necessary materials, information on the steps of the heel prick procedure, and general principles to be observed.

Stage 2: The students in the experimental and control groups were divided into seven subgroups, each consisting of 6 or 7 students. Each small group in the experimental and control groups received a one-hour lesson on the heel prick procedure. During this time, students’ questions were answered. At this stage, the pretests, including the Introductory Information Form, the State-Trait Anxiety Inventory (STAI), and the Self-Efficacy-Sufficiency Scale (SESS), were administered to the students. Each subgroup then received information about session objectives, confidentiality, and voluntary participation during a 10-minute prebriefing. Each student in the experimental and control groups was then asked to perform the heel prick procedure. Each student was given 20 min to perform the heel prick procedure. Learning objectives included performing the procedure using aseptic technique, following the correct procedure steps, selecting the correct prick point, and collecting an adequate blood sample. To ensure consistency across sessions, a standard scenario script (term infant, uncomplicated delivery, female baby, 3 kg and 50 cm.) and a 20-item skills checklist with validated reliability were used. Data for the experimental group were collected in a high-fidelity simulation environment with a remote/panel-based facilitator, while data for the control group were collected in a simulation laboratory with face-to-face facilitator support. In the experimental group, the facilitator provided support to the student from behind the control panel. In the control group, the facilitator provided face-to-face support to the student. The facilitator maintained the flow during the scenario by providing brief prompts only when necessary and did not make direct instructional interventions that could have affected the participants’ performance. Following the simulation, each subgroup received a 15-minute debriefing. The skill assessment forms completed by the researcher were used for further assessment. Any deficiencies or errors in the students’ skills or steps were corrected. Students were then asked to complete the State-Trait Anxiety Inventory (STAI), the Self-Efficacy-Sufficiency Scale (SESS), and the Student Satisfaction and Confidence in Learning Scale (SSCLS).

### Data collection tools

The data collection tools used to collect the research data are as follows.

#### Introductory information form

The introductory information form prepared by the researchers of 6 questions. The form included questions regarding the participants’ age, grade level, type of high school from which they graduated, employment status, family income, and their willingness to choose the profession.

#### Skill checklist

The heel prick blood collection skill (20 tasks/steps) is based on checklists for assessing student management, the Ministry of Health and WHO recommendations, training materials, and guidelines [21-23]. Each task/step was rated as “outstanding” if performed fully and correctly, and “needs improvement” if performed incompletely and incorrectly. The score is 1 point for “needs improvement” and 2 points for “satisfactory”. The skills checklist yields a minimum score of 1 and a maximum score of 40. The skill checklist was prepared using guidelines. Three expert opinions were also obtained (one public health specialist, one child health specialist, and one midwife). Furthermore, only reliability was examined for the skill checklist. The Kuder-Richardson 20 value was found to be 0.76.

The Introductory Information Form and Skill checklist used in this study were created by the researchers for this study (presented in the Appendix).

#### State-trait anxiety inventory (STAI)

The scale is a self-assessment questionnaire used to determine state anxiety levels. It was adapted into Turkish by Öner and Le Compte in 1985. The State Anxiety Inventory consists of a total of 20 items in scale. The State Anxiety Inventory requires the individual to describe how he/she feels at a certain moment and under certain conditions. It is a person’s perception of situations as stressful in general. The scale yields a total score that ranges from 20 to 80. A high score on the scale indicates a high level of anxiety, while a low score indicates a low level of anxiety [24]. In this study, the State Anxiety Inventory Cronbach Alpha value was found to be 0.92.

#### Self-efficacy-sufficiency scale (SESS)

The SESS adapted into Turkish by Gözüm and Aksayan [25], was designed to assess behavior and behavioral changes. The reliability and validity of the Turkish version of the scale were found to have a Cronbach’s Alpha internal consistency coefficient of 0.81 for the same sample. A total score between 23 and 115 can be obtained from the scale. A higher total score indicates a higher level of perceived self-efficacy. In this study, the Cronbach’s Alpha value was found to be 0.87.

#### Student satisfaction and self-confidence in learning scale (SSCLS)

This scale adapted into Turkish by Ünver et al. [26] was the total number of items to 12. The scale uses a 5-point Likert-type format and consists of two subdimensions: “Satisfaction with Current Learning” and “Confidence in Learning”. Higher total scores indicate greater student satisfaction and confidence in learning. The Cronbach’s Alpha value is 0.89. In this study, the Cronbach’s Alpha value was found to be 0.98.

### Ethics approval

This study was approved by Manisa Celal Bayar University Faculty of Medicine Health Sciences Ethics Committee (No: 20.478.486–1700). The students were informed about this study, and written and verbal informed consent was obtained from all students. The study remained faithful to the Helsinki Declaration on Human Rights throughout the study as the use of the human phenomenon and the protection of individual rights are essential.

### Data evaluation

Statistical analyses were conducted using IBM SPSS Statistics, Version 21.0 [27]. Descriptive statistics are presented as numbers and percentages for categorical data and mean for continuous data. The Chi-square test and Student’s t-test were employed for intergroup evaluations, while the paired sample t-test was utilized to compare changes within the groups over time. The Kuder-Richardson 20 value was used in the reliability analysis of the skill checklist.

## Results

Table 1 shows the sociodemographic characteristics of the students who participated in the study. The mean total age was 22.46 ± 2.22 years. It was observed that 71.7% of the students in the high-fidelity simulation group were 21–22 years old, 76.1% were high school graduates, 89.1% were unemployed, and 84.8% preferred the midwifery department intentionally. It was determined that 67.4% of the simulation laboratory group was in the 21–22 age group. 82.6% of the students graduated from high schools. It was noted that 87.0% of the students were unemployed and 80.4% of them preferred the midwifery department willingly (Table 1).

**Table 1:** Comparison of the descriptive characteristics of the group

Variables		Experimental Group		Control Group		x^2^/ *p*
		*n*	%	*n*	%	
Age X̄±Ss (Min/Max) 22.46 ± 2.22 (21/32)	21–22	33	71.7	31	67.4	0.205 / 0.650
	23 and above	13	28.3	15	32.6	
High school graduated from	High school	36	78.3	38	82.6	0.038/ 0.846
	Health vocational high school	8	17.4	6	13.0	
	Other	2	4.3	2	4.3	
Working status	Yes	5	10.9	6	13.0	0.103/ 0.748
	No	41	89.1	40	87.0	
The situation of choosing midwifery willingly	Yes	39	84.8	37	80.4	0.303/ 0.582
	No	7	15.2	9	19.6	
Total		46	100.0	46	100.0	

When the Skill Checklist total scores of the high-fidelity simulation group and the simulation group were evaluated, no significant difference was found between the two groups (t=-0.447, *p* = 0.887). In the skills checklist, a significant difference was found between the two groups in the procedure steps “Can relax his/her hand” (x^2^ = 4.039, *p* = 0.044), and “Knows to re-prick the heel with the lancet if there is a decrease in the amount of blood drops” (x^2^ = 12.132, *p* = 0.000, Table 2).

**Table 2:** Evaluation and comparison of heel Prick blood collection skills of the experimental and control groups

	Experimental Group				Control Group				Test / *p* value
	Needs to improve		Sufficient		Needs to improve		Sufficient		
	*n*	%	*n*	%	*n*	%	*n*	%	
1. Can prepare the materials.	1	2.2	45	97.8	-	-	46	100.0	**-**
2. The blood sample can fill in all the information on the filter paper.	3	6.5	43	93.5	4	8.7	42	91.3	1000*/500
3. Can give appropriate position to the baby.	1	2.2	45	97.8	-	-	46	100.0	**-**
4. It can determine the area where a heel blood sample can be taken.	1	2.2	45	97.8	-	-	46	100.0	-
5. It may gently warm the heel area for a few minutes.	3	6.5	43	93.5	5	10.9	41	89.1	0.714*/0.357
6. It can clear the area.	2	4.3	44	95.7	3	6.5	43	93.5	1000*/0.500
7. With the thumb and index finger forward, the heel can be squeezed.	2	4.3	44	95.7	-	-	46	100.0	**-**
8. The other 3 fingers can be rubbed behind.	1	2.2	45	97.8	1	2.2	45	97.8	1.000*/0.753
9. She can stroke the heel three times	3	6.5	43	93.5	5	10.9	41	89.1	0.714*/0.357
10. The heel can be pierced once with a lancet at a suitable place.	1	2.2	45	97.8	1	2.2	45	97.8	1.000*/0.753
11. She can relax his/her hand	**8**	**17.4**	**38**	**82.6**	**2**	**4.3**	**44**	**95.7**	4.039*/0.044
12. It can wipe away the first drop of blood.	2	4.3	44	95.7	2	4.3	44	95.7	1000*/0.692
13. It can form a large, thick drop of blood that can be collected on filter paper.	1	2.2	45	97.8	-	-	46	100.0	**-**
14. It can make the drop fill the entire ring on the filter paper with a single touch.	1	2.2	45	97.8	-	-	46	100.0	**-**
15. Only one surface of the filter paper can be used for blood collection.	1	2.2	45	97.8	-	-	46	100.0	**-**
16. It can also fill the remaining loops on the filter paper.	1	2.2	45	97.8	-	-	46	100.0	-
17.Knows to re-prick the heel with the lancet if there is a decrease in the amount of blood drops	**1**	**2.2**	**45**	**97.8**	**13**	**28.3**	**33**	**71.7**	12.132*/0.000
18. After the procedure is completed, you can press the heel.	1	2.2	45	97.8	-	-	46	100.0	-
19. You can dry the filter paper from which the blood sample was taken on a dry, clean, flat and horizontal surface.	1	2.2	45	97.8	2	4.3	44	95.7	1.000*/0.500
20.The filter paper can be kept in an envelope or box until it is sent to the relevant center.	1	2.2	45	97.8	1	2.2	45	97.8	1000*/0.753
Total score (**X̄±Ss**)	39.21 ± 2.96				39,15 ± 0,91				0.447** / 0,887

^*^ Fisher’s exact test

^**^ Student’s t-test

Evaluation of the STAI state total scores in both groups revealed that there was no significant difference between the pre-test scores (t=-0.121, *p* = 0.904), whereas there was a significant difference between the post-test scores, with the high-fidelity simulation group having a significantly higher score (t = 2.676, *p* = 0.009). Moreover, it was found that the post-test score of the high-fidelity simulation group (t=-2.033, *p* = 0.048) was significantly higher than the pre-test score (*p* < 0.05, Table 3).

**Table 3:** STAI pre-test and post-test total score distributions and comparison of experimental and control groups

	Experimental Group		Control Group		Significance test	Effect size
	*n*	X̄±Ss	*n*	X̄±Ss	t* / *p*	
Pre-test	46	44.28 ± 5.41	46	44.43 ± 6.60	-0.121 / 0,904	-
Post-test	46	46.47 ± 5.31	46	43,36 ± 5,81	**2.676 / 0**,**009**	**0.600**
Significance test	t**:-2.033 p: 0,**048**		t**: 1.069 p: 0,291			

t* Student’s t-test

t** Paired Sample t-test

When the SESS total scores of the high-fidelity simulation group and the simulation group were evaluated, no significant difference was found between the pre-test and post-test scores within and between the groups (*p* > 0.05, Table 4).

**Table 4:** SESS pre-test and post-test total score distributions and comparison of experimental and control groups

	Experimental Group		Control Group		Significance test
	*n*	X̄±Ss	*n*	X̄±Ss	t */ *p*
Pre-test	46	85.54 ± 12.94	46	82.84 ± 12.62	1.011 / 0,315
Post-test	46	84.08 ± 10.94	46	82,69 ± 9,92	0.638 / 0,525
Significance test	t**:-1.002 p: 0,322		t**: 0.097 p: 0,923		

t* Paired Sample t testi

t** Student t testi

When the SCLS total scores of the high-fidelity simulation group and the simulation group were evaluated, it was revealed that the post-test score of the high-fidelity simulation group was significantly higher (t = 2.372, *p* = 0.020, Table 5).

**Table 5:** SSCLS total score distributions and comparison of experimental and control groups

	Experimental Group		Control Group		Significance test	Effect size
	*n*	X̄±Ss	*n*	X̄±Ss	t / *p*	
SCLS total score	46	4.66 ± 0.52	46	4,28 ± 0,92	**2.372 / 0**,**020**	**0.451**
Satisfaction with Current Learning Subscale	46	4.62 ± 0.57	46	4,27 ± 0,95	**2.144 / 0**,**035**	
Learning Self-Confidence Subscale	46	4.68 ± 0.51	46	4,30 ± 0,92	**2.466 / 0**,**016**	

t: Student t testi

## Discussion

In this study, the effects of simulation environments used by midwifery students on their skills in heel prick blood sampling in newborns, learning satisfaction, anxiety, and self-efficacy were evaluated. Simulation facilitates the link between theory and practice, thereby enhancing students’ learning ability. Various quantitative studies in the literature have highlighted that high-fidelity simulation practices contribute more significantly to skill acquisition compared to low-fidelity applications, especially by improving communication with patients [7, 28]. Other studies, however, have found that while high-fidelity simulation applications lead to an increase in students’ knowledge scores, no significant improvement in skill levels was observed [29, 30]. In this study, no significant difference was found between the skill scores of students in the high-fidelity simulation and simulation laboratory groups (*p* > 0.05). The results are consistent with the literature. It is clear that simulation laboratories contribute to skill development regardless of the level of fidelity and the face-to-face/remote role of the facilitator, and the learning rate accelerates in such environments.

Providing safe and high-quality care for newborn babies is one of the core responsibilities of midwives [31]. However, in midwifery education, it is not always possible for students to gain appropriate clinical experiences due to limited clinical practice areas, shorter hospital stays, and concerns such as malpractice. In this context, the selection of appropriate simulators for teaching and developing midwifery skills is crucial for the quality of education. Therefore, high-fidelity simulation environments that offer students the opportunity to experience and manage real clinical situations in a safe environment are preferred [2, 32–34]. Recent research has reported that high-fidelity simulation practices used in midwifery education increase students’ satisfaction with their learning experience and reduce anxiety [35–37]. However, some studies also report that anxiety increases depending on the scenario, student characteristics, and simulation design factors. These studies report that students’ mean STAI-state scores ranged between 33.45 and 48.24 and were above the average [9, 38]. In this study, students in both simulation groups were also found to have high anxiety scores. The STAI-state score of students practicing in the high-fidelity simulation environment (46.47) was found to be higher than the score of students working in the simulation laboratory (43.36).

Furthermore, in studies, the satisfaction scores of students practicing in high-fidelity simulation environments range between 4.32 and 4.82. Students expressed satisfaction with the simulation application, stating that they felt the simulation provided a safe learning space for making and learning from mistakes [19, 30, 39]. In this study, students’ satisfaction and self-confidence in learning scores were found to be higher in the high-fidelity simulation environment (4.66) compared to the simulation laboratory group (4.28). When the sub-dimensions of satisfaction and self-confidence in learning were examined, the scores of students practicing in the high-fidelity simulation environment (satisfaction = 4.62; self-confidence = 4.68) were found to be higher than the scores of students practicing in the simulation laboratory (satisfaction = 4.27; self-confidence = 4.30). The co-existence of high learning satisfaction with high anxiety in a high-fidelity simulation environment may seem paradoxical. However, this situation is actually expected. This is because “realism” in high-fidelity simulation environments is addressed in three main dimensions: physical environment, functional (compatibility of the equipment and scenario with the real clinical function), and psychological (the student’s feeling of “being real”). The student’s feeling of “being real” is particularly a determinant of the stress level [40, 41]. This stress usually stems from the student managing the process alone in the “real-like” simulation environment, the fear of making mistakes, time pressure, and the feeling of being observed by the facilitator. Even though students experience the fear of making mistakes, knowing that it is not on a real patient increases self-efficacy and satisfaction after the stress is resolved [42-44]. In this research, it is thought that the stress of the student, who was left alone with the facilitator behind the control panel, increased due to the complete simulation of a hospital room in the high-fidelity simulation environment. Students’ learning satisfaction is not only affected by the level of stress but also by how the stress is managed. Especially the debriefing process is a critical factor in managing stress. Debriefing allows students to make sense of the stress they experienced and turns negative emotions into positive learning outcomes. Therefore, an appropriately structured debriefing process mitigates the negative effects of stress, ensuring that students are satisfied with the experience and feel more self-efficacious in similar situations in the future [35, 45]. We believe that the student managing the heel prick blood sampling process alone in the high-fidelity simulation environment and the facilitator being behind the control panel were effective in the higher satisfaction score.

Simulation also increases students’ self-efficacy through repeated practice. Bandura stated that educational systems play a key role in shaping self-efficacy beliefs [46]. Although it is assumed that different teaching methods can increase self-efficacy, no significant difference was found in self-efficacy scores between groups or within groups between pre- and post-tests in this study (*p* > 0.05). Critical situations rarely encountered in traditional clinical or professional settings can be practiced repeatedly in a safe simulation environment. As students successfully complete scenarios and overcome challenges, they gain concrete evidence of their abilities. These tangible successes are the fundamental factors that strongly and permanently increase self-efficacy [47]. The lack of difference in heel prick blood sampling skills between students in both simulation groups in this study explains their similar self-efficacy.

### Limitations

This study has several limitations. First, data collection were carried out by the same researcher for all groups, potentially introducing a bias in terms of objectivity. Second, the study’s data were collected at a single institution, and the sample size was small, limiting the generalizability of the findings to the broader community.

## Conclusion

In this study, students’ heel prick skills and self-efficacy were found to be similar regardless of the fidelity level of the simulation laboratories and the role of the facilitator. In the high-fidelity simulation environment/facilitator panel, students’ anxiety and satisfaction levels were higher. As a result of this study, it may be recommended to increase the use of high-fidelity simulation environments in midwifery education, to use more comprehensive simulation training that includes longer and more varied skill exercises, and to evaluate the outcomes.

## Supplementary Information

Supplementary Material 1.

## Data Availability

The datasets used and/or analysed during the current study are available from the corresponding author on reasonable request.
