# Supplementary material for: The effect of simulation training on midwifery students’ skills, satisfaction with learning, anxiety, and self-efficacy in neonatal heel prick blood collection
Source: BMC Med Educ. 2025 Dec 27;26:314. doi: 10.1186/s12909-025-08303-3 (PMC12918260; doi:10.1186/s12909-025-08303-3)
Supplement: Supplementary file 1 — Supplementary Material 1. [file 12909_2025_8303_MOESM1_ESM.docx]

**The Effect of Simulation Training on Midwifery Students' Skills, Satisfaction with Learning, Anxiety, and Self-Efficacy in Neonatal Heel Prick Blood Collection**

**A-Introductıonary Informatıon Form**

1. Your age ………………

2. Which high school did you graduate from?

□ Regular High School

□ Anatolian High School

□ Health Vocational High School

□ Other (please specify) …………

3. Do you work at any job? □Yes (please specify) ……………….. □ No

4. Did you choose your profession willingly? □Yes □ No

**B-Skill Checklist Form**

|  | **Needs improvement**  **(1 point)** | **Satisfactory**  **(2 point)** |
| --- | --- | --- |
| 1. Can prepare the materials. |  |  |
| 2. The blood sample can fill in all the information on the filter paper. |  |  |
| 3. Can give appropriate position to the baby. |  |  |
| 4. It can determine the area where a heel blood sample can be taken. |  |  |
| 5. It may gently warm the heel area for a few minutes. |  |  |
| 6. It can clear the area. |  |  |
| 7. With the thumb and index finger forward, the heel can be squeezed. |  |  |
| 8. The other 3 fingers can be rubbed behind. |  |  |
| 9. She can stroke the heel three times |  |  |
| 10. The heel can be pierced once with a lancet at a suitable place. |  |  |
| 11. She can loosen her grip |  |  |
| 12. It can wipe away the first drop of blood. |  |  |
| 13. It can form a large, thick drop of blood that can be collected on filter paper. |  |  |
| 14. It can make the drop fill the entire ring on the filter paper with a single touch. |  |  |
| 15. Only one surface of the filter paper can be used for blood collection. |  |  |
| 16. It can also fill the remaining loops on the filter paper. |  |  |
| 17.If the amount of blood drops decreases, she knows that she can pierce the heel with the lancet again. |  |  |
| 18. After the procedure is completed, you can press the heel. |  |  |
| 19. You can dry the filter paper from which the blood sample was taken on a dry, clean, flat and horizontal surface. |  |  |
| 20.The filter paper can be kept in an envelope or box until it is sent to the relevant center. |  |  |

The references for the State-Trait Anxiety Inventory, Self-Efficacy-Sufficiency Scale, Student Satisfaction, and Self-Confidence in Learning scales are indicated in the text.
